# Pediatric Hospital Acquired Venous Thromboembolism

**DOI:** 10.3389/fped.2017.00198

**Published:** 2017-09-19

**Authors:** Char M. Witmer, Clifford M. Takemoto

**Affiliations:** ^1^Department of Pediatrics, Division of Hematology, Children’s Hospital of Philadelphia, Philadelphia, PA, United States; ^2^Pediatric Hematology, The Johns Hopkins University, Baltimore, MD, United States

**Keywords:** pediatric, venous thromboembolism, hospital acquired, prevention, central venous catheter

## Abstract

Pediatric hospital acquired venous thromboembolism (HA-VTE) is an increasing problem with an estimated increase from 5.3 events per 10,000 pediatric hospital admissions in the early 1990s to a current estimate of 30–58 events per 10,000 pediatric hospital admissions. Pediatric HA-VTE is associated with significant morbidity and mortality. The etiology is multifactorial but central venous catheters remain the predominant risk factor. Additional HA-VTE risk factors include both acquired (recent surgery, immobility, inflammation, and critical illness) and inherited risk factors. Questions remain regarding the most effective method to assess for HA-VTE risk in hospitalized pediatric patients and what preventative strategies should be implemented. While several risk-assessment models have been published in pediatric patients, these studies have limited power due to small sample size and require prospective validation. Potential thromboprophylactic measures include mechanical and pharmacologic methods both of which have associated harms, the most significant of which is bleeding from anticoagulation. Standard anticoagulation options in pediatric patients currently include unfractionated heparin, low molecular weight heparin, or warfarin all of which pose a monitoring burden. Ongoing pediatric studies with direct oral anticoagulants could potentially revolutionize the prevention and treatment of pediatric thrombosis with the possibility of a convenient route of administration and no requirement for monitoring. Further studies assessing clinical outcomes of venous thromboembolism (VTE) prevention strategies are critical to evaluate the effectiveness and harm of prophylactic interventions in children. Despite HA-VTE prevention efforts, thrombotic events can still occur, and it is important that clinicians have a high clinical suspicion to ensure prompt diagnosis and treatment to prevent further associated harms.

## Epidemiology

Hospital acquired venous thromboembolism (HA-VTE) is currently considered the second most common contributor to harm in hospitalized pediatric patients secondary only to central line-associated infection (1). It is a rapidly increasing problem, with an estimated increase from 5.3 events per 10,000 pediatric hospital admissions in the early 1990s to a current estimate of 30–58 events per 10,000 pediatric hospital admissions (2–5). The pathogenesis of venous thromboembolism (VTE) is associated with the three main elements described in Virchow’s triad, including stasis of blood flow, endothelial injury, and hypercoagulability, and commonly arises as a result of concurrent risk factors. While the pathogenesis of VTE in pediatric patients is multifactorial, the presence of a central venous catheter (CVC) remains the single most important risk factor (3, 6, 7).

The age of onset for pediatric VTE is bimodal, revealing peaks in the neonatal and adolescent age groups (2). The overall frequency of VTE in the adult population (inpatient and outpatient setting) remains significantly more common than children with an incidence as high as 1 in 100 individuals older than 80 years versus 1 in 100,000 pediatric patients (8, 9). VTE in hospitalized adult patients remains a significant cause of morbidity and mortality and concerted efforts have been made to identify VTE risk factors and to develop effective prevention strategies (10, 11). While less frequent, VTE in hospitalized children also has been recognized as major contributor to harm and effective interventions are needed (1).

The resultant harms in pediatric patients from VTE are numerous and range from death to pulmonary embolism, paradoxical emboli, infection, post thrombotic syndrome, loss of venous access, and pain at the site of thrombosis. Pediatric HA-VTE has been associated with increased length of stay and cost (12). The estimated mortality rate associated with pediatric VTE is 2.2% (6).

## Risk Factors for HA-VTE

Observational, case–control and non-case–control studies in both adults and children have identified a number of VTE risks in hospitalized patients (13). These risks may be either acquired (such as surgery, immobility, inflammatory conditions, CVCs) or inherited (such as Factor V Leiden, prothrombin gene mutation, anticoagulant deficiency). In general, most pediatric patients with a HA-VTE have multiple VTE risk factors present at the time of the thrombotic event.

Recently, Mahajerin et al. performed a meta-analysis of the published studies for VTE risk factors in hospitalized pediatric patients. The authors found that the presence of central catheters, increased length of stay, intubation, and ICU admissions were significantly associated with increased odds ratios for HA-VTE (13). The relative paucity of studies in pediatric patients highlights the challenges of obtaining high quality evidence for this rare event and warrants further research and multi-institutional trials. While a number of studies have demonstrated specific medical conditions, surgical interventions and age as risks for HA-VTE, the low incidence of HA-VTE in children has reduced the power of these studies to detect other potentially relevant clinical risks. We review the current evidence for HA-VTE risks below.

### Age

As previously mentioned, the overall incidence of VTE in children is bimodal; there is a peak in infants with subsequent decline in infancy and childhood. The incidence then increases in adolescence and continues to increase throughout adulthood (2, 5, 14). In adults the incidence continues to rise with age and is 10 to 100 times higher than in children (9). The progressive increase in VTE that starts in early adolescence is likely multifactorial. Physiologic changes such as increased FVIII and von Willebrand activity may contribute to increased risk in adults, as well as the use of estrogens in females. In addition, the higher VTE incidence may also reflect the increase in comorbid conditions, such as renal disease, malignancy, and trauma, which are more common in adults (4, 5). Conversely, neonates have a high rate of HA-VTE but this is most likely in large part secondary to the use of CVC’s in this critically-ill population, as the rate of CVC-associated VTE is increased in this age group (5).

### Mobility

Reduced mobility as a VTE risk factor is well established in the adult population, and some studies in children have also demonstrated this risk (13). However, the details of degree and chronicity of immobility in these studies are not reported. In adult studies, definitions vary considerably (15); some have used bed rest or out of bed for <30 min per day (16). Acute flaccid paralysis has been shown to be a major risk factor for the development of VTE in adults (17). The VTE risk of chronic immobility in children with quadriplegia is less clear. Additionally, the notion that venous stasis is a VTE risk factor in an infant who is not walking is doubtful. Likely, the combination of acute immobility in a hospitalized adolescent patient is a VTE risk, similar to what is seen in adults. A study by Branchford et al. demonstrated that intubation is a risk for HA-VTE, which may be a surrogate for immobility (18).

### Medical Conditions

A number of chronic diseases have an increased incidence of VTE, including active malignancy, congenital cardiac disease, renal disease, and rheumatologic disorders (2, 5, 14). Besides the frequent presence of CVCs in patients with these diagnoses, common contributor for VTE risk include acute inflammation, which can result in increases in prothrombotic factors, such a FVIII, vWF, and fibrinogen. Several studies have shown that systemic infection is also a significant VTE risk factor; the mechanisms by which infection contributes to thrombosis are multifactorial (18–20). Infection can be associated with upregulation of prothrombotic factors; in addition, structures called neutrophil extracellular traps can be formed in response to infection and inflammation and are implicated in thrombogenesis (21). Acquired thrombophilia may contribute to risk in hospitalized patients, such as acquired antithrombin deficiency with nephrotic syndrome, a draining chylous effusion or with asparaginase therapy for leukemia. Additional risk factors for HA-VTE in children include obesity and estrogens (18, 22). Overall, medical complexity is a risk as those diagnosed with multiple diseases have an increased odd of VTE (5, 18).

### Surgery, Trauma, and Intensive Care

Surgery is a known risk for VTE; this is likely secondary to a combination of factors including a post-surgical inflammatory state, immobility, and CVCs. In particular, some procedures, such as orthopedic surgery is associated with significant risk in adults. However, the incidence of VTE in children with orthopedic procedure appears to be significantly lower for unclear reasons (23, 24). For patients undergoing cardiac surgery, the overall prevalence of thrombosis is significant at 11% (25) with catheter-associated thrombi in the order of 15% (26). The high VTE rate in these patients may be contributed by stasis and turbulent blood flow associated with congenital heart disease, and by platelet activation with the cardiac bypass circuit. Pediatric trauma patients are also at increased risk; VTE has been shown to be associated with an increased injury severity score, surgery, and blood transfusion (27, 28). There is a significantly higher incidence and risk of VTE in pediatric patients admitted to intensive care units. This finding likely reflects the medical complexity of these patients as well as the other associated risk of CVCs and surgery in this population (13).

### Catheters

Central venous catheters are the most common risk associated with VTE in children. The characteristic of CVC placement include location (upper versus lower extremity), type [peripherally placed catheters (PICC) versus implanted], duration, and catheter size. Based on current data, it is unclear if the incidence of VTE with PICCs is greater than with tunneled catheters and prospective studies are needed (29, 30). In surveillance studies, the incidence of VTE associated CVCs placed in the PICU is significant, and has been reported to be 18% (31). While CVC’s are more frequently placed in the intensive care units, it is a risk factor in non-ICU settings as well (5, 19, 20). A major problem with catheter-related thrombosis in children is with the size of catheters compared to vessel size. An optimal diameter ratio of 1:3 has been suggested; however, this may not be achievable in infants and small children (32). These physical restraints add to the challenge of VTE prevention with CVCs in this population.

### Inherited Thrombophilia

The presence of an inherited thrombophilia, such as a deficiency of antithrombin, protein C or S, or the Factor V Leiden and prothrombin mutations, is associated with an increased odds of developing VTE (33). The odds of VTE are further increased with greater than two genetic traits suggesting that risks are additive. The presence of thrombophilia has been used in risk-assessment models (RAMs) in adults (10) as well as pediatric acute leukemia (34). However, its utility for predicting HA-VTE in the pediatric population has not yet been demonstrated.

## VTE Risk Assessment

With recent incidence estimates of all HA-VTE diagnosis between 1 in 141 and 532 admissions (14, 35), it is critical to target VTE prophylaxis strategies to only those patients at highest risk for developing VTE. Likewise, low-risk patients need to be identified to avoid exposure to the bleeding risks of anticoagulation and/or the cost and challenges of intermittent pneumatic compression device (IPC). The principal that the VTE development in hospitalized pediatric patients is multifactorial and that the strengths of the VTE risks vary lend this outcome to the use of RAMs. RAMs have been developed and validated for hospitalized adults, but few have been published for pediatrics (10, 36, 37). With the mandate imposed by 2014 Joint Commission on Accreditation of Healthcare Organizations for VTE risk assessment screening on all hospitalized adults (18 years and older), awareness of HA-VTE in children has been heightened, and the need for improved screening tools has been recognized (1).

Four RAMs based on risk factor identification for the development of VTE in hospitalized children have been published (Pediatric inpatients, Table 1) (18–20, 35). In these models, three to six independent risks factors were identified in case–control studies and a weighted score was assigned to the risk to derive a score. Common factors to all the models were increased length of stay and infection. In addition, the presence of CVCs and intubation/immobility were identified in more than one RAM. Several of these factors in children are also incorporated in validated adult RAMs, such as infection and immobilization (10, 36). However, older age, history of thrombophilia, and malignancy which were identified in adult RAM models were not found in these studies in children (10, 36, 37). The pediatric studies are challenged by small sample sizes, retrospective study design and lower incidence of VTE, which limit the power to detect potentially significant risk factors. Additionally, many of these models included length of stay which cannot be used in a prospective manner for VTE risk prediction. It should be noted that two RAMs in children with trauma and one in pediatric patients with acute lymphoblastic leukemia who have a high risk of VTE have also been developed and validated (Table 1); these RAMs were developed similarly by identification of VTE risks and weighting of factors based on odds ratios. These models have been validated in independent cohorts, but will need to be studied prospectively to determine their utility (28, 34, 38).

**Table 1:** Pediatric risk-assessment models (RAMs) for hospital acquired venous thromboembolism.

RAM	Patient population	Number of factors	VTE risk factors (maximum points)	Comments
**Pediatric inpatients**				
Colorado Children’s Hospital (18)	Medical/ICU (age 0–21 years)	3	Intubation, infection, LOS ≥ 5days	3.6% probability of VTE with 3 factors
Peds-Clot Riley Hospital for Children and Children’s Memorial Hospital (35)	Medical/ICU (age 0–20 years)	6	Immobilization (3), direct ICU admission (0.5), CVC (1), blood stream infection (1), OCP (2), LOS ≥ 7 days (2)	9.5 point risk score; Score of 3: sensitivity: 57–70%; specificity: 80–88% AUC: 0.852–0.89
Johns Hopkins All Children’s Hospital (20)	Medical/Non-ICU (age 0–21 years)	3	CVC (5), infection (2), LOS ≥ 4 days (1)	8 point risk score; 8 points: 12.5% VTE; 7 points 1.1% VTE; ≤6 points 0.1% VTE
Johns Hopkins All Children’s Hospital (19)	ICU, non-cardiac (age 0–21 years)	3	CVC (8), infection (1), LOS ≥ 4 days (6)	15 point risk score; 15 points: 8.8% VTE; 7–14 points 1.3% VTE; ≤7 points 0.03% VTE
**Pediatric trauma**				
ROCKiT (Johns Hopkins Hospital trauma registry and National Trauma Data Bank) (28)	Trauma (age 0–21 years)	6	Older age (4), intubation (4), high ISS (7), low GCS (1), surgery (5), blood transfusion (2)	23 point risk score; score of 13: sensitivity: 87%; specificity: 81%; AUC: 0.9
National Trauma Data Bank (38)	Trauma (age 0–17 years)	10	Older age (147), female sex (4), ICU admission (171), intubation (97), low GCS (34), CVC (61), pelvic fracture (33), lower extremity fracture (36), major surgery (150), blood transfusion (58)	797 point risk score; >688 points: >5% VTE; 524–688 points 1–5% VTE; ≤523 points <1% VTE; AUC: 0.945
**Pediatric malignancy**				
BFM/COALL/FRALLE acute leukemia protocol (34)	Acute lymphoblastic leukemia in induction therapy (age 1–18 years)	3	steroid/asparaginase (1), CVC (1), thrombophilia (2)	Maximum score range (3–4) depended on treatment protocol >2.5 points: 64.7% VTE ≤2.5 points 2.5% VTE

*VTE, venous thromboembolism; ICU, intensive care unit; LOS, length of stay; CVC, central venous catheter; OCP, oral contraceptive pill; AUC, area under the curve; ISS, injury severity score; GCS, Glasgow Coma Scale; BFM, Berlin-Frankfurt-Münster 90/95/2000; COALL, Cooperative Acute Lymphoblastic Leukemia 92/95; FRALLE, French Acute Lymphoblastic Leukemia 2000*.

Risk-assessment models can be used to stratify VTE risk in order to better balance the risks and benefit of the prophylactic options. It has been suggested in adults that a population with a VTE frequency of 2% or higher should be targeted for pharmacologic prophylaxis, and populations with risks between 1 and 2% would be appropriate for mechanical prophylaxis (37). While VTE occurrence is reduced by a half with heparin prophylaxis in adults, these data are lacking in pediatrics. The benefits of prophylaxis must be weighed against the risks. In children, the bleeding risk for prophylactic low molecular weight heparin (LMWH) has been reported to be 0.8% for major bleeding and 3% for minor bleeding (39). The uncertainties of the risk/benefits of interventions in children add to the challenges of implementation of RAMs and require additional studies.

## Prevention: Mechanical and Pharmacological

Venous thromboembolism prevention strategies for hospitalized pediatric patients include early mobilization, mechanical, and/or pharmacologic prophylaxis. In an effort to minimize harm, the strategies generally have utilized early mobilization and mechanical prophylaxis in patients determined to be at moderate risk for VTE and pharmacologic interventions are reserved for those patients with the highest VTE risk (40, 41).

### Early Mobilization

Encouraging maximal mobility of all hospitalized patients is a relatively simple approach for VTE prevention. Movement of the calf muscle with ambulation prevents venous stasis in the lower extremities which as previously discussed is one of the key risk factors for VTE development. Maximal activity should be encouraged no matter the patient’s VTE risk. In addition, this strategy may also have other benefits. In adult studies, implementation of early and maximal mobility has been associated not only with decreased VTE occurrence but also decreased length of stay and improved cognitive and functional outcomes (42, 43).

### Mechanical Prophylaxis

Mechanical prophylaxis includes the use of either IPCs or graduated compression stockings. Compression stockings provide circumferential pressure that gradually decreases from the ankle to the thigh. IPCs utilize intermittent inflation and deflation of a “sleeve” to increase venous return from the lower extremities mimicking that action of the calf muscles. In addition, IPCs have been demonstrated to activate systemic fibrinolysis which could theoretically promote clot dissolution (44–50).

Currently, there are no pediatric trials assessing the effectiveness of mechanical prophylaxis. Adult studies support the efficacy of mechanical interventions in preventing DVT and PE in a number of different clinical situations including post trauma, post-surgical, and the medically ill hospitalized patient (51–55). Until recently, questions remained regarding the efficacy of IPCs versus compression stockings. A recent prospective study of adult ICU patients compared the incidence of VTE in those patients receiving either IPC or compression stockings. Only IPC, and not compression stockings, was associated with a lower VTE incidence as compared with controls [0.45 (95% CI 0.22–0.95)] (51). In addition, a large meta-analysis in hospitalized medical patients also supported the finding that IPC is superior to compression stockings in preventing DVT (54).

### Pharmacologic Prophylaxis

There are limited studies addressing efficacy and safety of anticoagulation (pharmacologic prophylaxis) for VTE prevention in pediatric patients. The 2012 Chest guidelines provide recommendations for therapeutic ranges for prophylactic anticoagulation (warfarin INR 1.3–1.9 or LMWH anti-Xa 0.1–0.3 U/mL) (56). They do not comment on indications for VTE prophylaxis in hospitalized pediatric patients (56). Much of what is currently used in pediatric patients is extrapolated from the adult literature, especially as it pertains to the adolescent patient with VTE risk factors that are similar to that of adults. There are numerous studies that have demonstrated efficacy of anticoagulation for reducing hospital acquired VTE in both surgical and non-surgical adult patients and its use is considered standard of care (11, 53). In pediatric patients at risk for VTE, anticoagulation with either a LMWH or subcutaneous unfractionated heparin (UFH) has been utilized for prophylaxis (40). While weight based dosing is used for LMWH, there are no weight based dosing guidelines for subcutaneous UFH which limits pediatric use. Low-dose UFH continuous infusions have also been utilized, primarily in the post-surgery setting, but published data on efficacy is lacking (26). Currently, direct oral anticoagulants are not FDA approved for pediatric use and clinical trials are underway to assess safety and efficacy for both VTE treatment and prophylaxis. The use of direct oral anticoagulants could potentially revolutionize the prevention and treatment of pediatric thrombosis with the possibility of a convenient route of administration and no requirement for monitoring.

### Central Venous Catheters

Unlike in adults, the most important risk factor for VTE in pediatric patients is the presence of a CVC. The 2012 CHEST guidelines recommend against primary prophylaxis after the placement of a central venous line (56). There are three randomized clinical trials that studied primary CVC prophylaxis in pediatric patients using prophylactic dosing of either LMWH (anti-Xa goal 0.1–0.3 U/mL), UFH (10 U/kg/h), or warfarin (INR goal 1.3–1.9) (26, 57, 58). None of these trials were able to demonstrate a difference in thrombotic events between the two treatment arms, although these studies were generally underpowered. Specifically, the number of subjects was too small to provide enough statistical power, and this was most commonly secondary to challenges with enrollment (56). A recent systematic review and meta-analysis of thromboprophylaxis in children was unable to find evidence that it reduced the risk of CVC-associated thrombosis (59). Ongoing research is needed to determine the most effective way to prevent CVC-associated thrombosis.

When considering prophylactic measures for VTE in hospitalized pediatric patients it is important to assess for potential harms from the intervention (60). Contraindications to mechanical prophylaxis include a device that does not fit the patient, distal peripheral IV access, skin, or lower extremity conditions that result in pain with compression (i.e., fracture, vaso-occlusive pain in a patient with sickle cell disease, burns, etc.) (60). Contraindications to anticoagulation include the presence of active bleeding, a concurrent coagulopathy (acquired or congenital), acute stroke, epidural catheter, uncontrolled severe hypertension, recent surgical procedure with a high risk of surgical site hemorrhage, or an intracranial mass (56).

## VTE Detection and Diagnosis

Despite prevention efforts, thrombotic events can still occur in hospitalized pediatric patients, and it is imperative that VTE remain a consideration when concerning clinical signs and symptoms are present. The signs and symptoms of VTE are dependent on the site and degree of venous occlusion. When an extremity is affected the clinical signs include swelling and pain of that extremity. If there is embolization of the thrombus the patient may develop a sudden onset of pleuritic chest pain, shortness of breath, and/or persistent tachycardia. A large PE can present as acute respiratory and cardiac failure. In a patient who is intubated and unable to report symptoms a PE could present as an acute respiratory decompensation. For those patients with an abnormal connection between the right and left side of the heart, a venous embolism could become a paradoxical embolism with resultant stroke or distal ischemia to the gut, kidneys or limbs.

When VTE is suspected, the imaging modality selected is dependent on the site of thrombosis. Historically, venography was the gold standard, but it has increasingly fallen out of favor, being replaced by other imaging modalities like ultrasonography, CT, or MR venography (61). The d-dimer has not been validated in pediatric clinical trials for the diagnosis of VTE, and has only been studied as a risk for recurrence, making interpretation difficult in this population (62).

Early recognition and diagnosis of a hospital acquired VTE will ensure prompt treatment with full anticoagulation which will minimize VTE associated harms. The goal of anticoagulation is to stop clot propagation, prevent embolism (pulmonary and paradoxical), preserve vascular access and prevent bacteremia (56). In the clinical setting of a lower extremity DVT and an absolute contraindication to anticoagulation, a temporary inferior vena cava filter should be considered for children who weigh greater than 10 kg (56). The filter should be removed as soon as the contraindication has resolved and anticoagulation can be instituted.

## Summary

While HA-VTE is uncommon in children compared to adults, it is increasing and associated with significant morbidity. Validated risk-assessment tools to identify adult patients at high risk for the development of VTE in the hospital are in widespread use. Some risk factors for VTE are shared between children and adults; however, there are significant limitations for the use of these tools in children. Importantly, several pediatric RAMs have been published, but the studies are relatively small and need prospective validation. Strategies to identify pediatric patients at highest risk for HA-VTE are needed to target interventions to prevent non-CVC-associated VTE. Further studies assessing clinical outcomes of VTE prevention strategies are critical to assess the effectiveness and harm of prophylactic interventions in children. A major challenge remains with CVC-associated VTE, since currently there is no evidence that pharmacologic prophylaxis, or other interventions, are effective for prevention.

## Author Contributions

CW and CT both equally contributed to the drafting of the original manuscript and reviewed and approved of the final submitted version.

## Conflict of Interest Statement

CT reports that he receives grant support for pharmaceutical trials from Novo Nordisk and Mast Therapeutics. CW has no conflict of interests to declare.

## References

[1] Witmer C Davis D Branchford BR Jaffray J Doellman D Raffini L Children’s Hospitals’ Solutions for Patient Safety Recommended Bundles: Adverse Drug Events and Venous Thromboembolism. (2016). Available from: http://www.solutionsforpatientsafety.org/wp-content/uploads/SPS-Recommended-Bundles.pdf

[2] Raffini L Huang YS Witmer C Feudtner C. Dramatic increase in venous thromboembolism in children’s hospitals in the United States from 2001 to 2007. Pediatrics (2009) 124:1001–8. 10.1542/peds.2009-0768 19736261

[3] Massicotte MP Dix D Monagle P Adams M Andrew M. Central venous catheter related thrombosis in children: analysis of the Canadian Registry of Venous Thromboembolic Complications. J Pediatr (1998) 133:770–6. 10.1016/S0022-3476(98)70149-0 9842042

[4] Andrew M David M Adams M Ali K Anderson R Barnard D Venous thromboembolic complications (VTE) in children: first analyses of the Canadian Registry of VTE. Blood (1994) 83:1251–7. 8118029

[5] Takemoto CM Sohi S Desai K Bharaj R Khanna A McFarland S Hospital-associated venous thromboembolism in children: incidence and clinical characteristics. J Pediatr (2014) 164:332–8. 10.1016/j.jpeds.2013.10.025 24332452

[6] Monagle P Adams M Mahoney M Ali K Barnard D Bernstein M Outcome of pediatric thromboembolic disease: a report from the Canadian Childhood Thrombophilia Registry. Pediatr Res (2000) 47:763–6. 10.1203/00006450-200006000-00013 10832734

[7] Richardson MW Allen GA Monahan PE. Thrombosis in children: current perspective and distinct challenges. Thromb Haemost (2002) 88:900–11. 12529737

[8] Stein PD Kayali F Olson RE. Incidence of venous thromboembolism in infants and children: data from the National Hospital Discharge Survey. J Pediatr (2004) 145:563–5. 10.1016/j.jpeds.2004.06.021 15480387

[9] Beckman MG Hooper WC Critchley SE Ortel TL. Venous thromboembolism: a public health concern. Am J Prev Med (2010) 38:S495–501. 10.1016/j.amepre.2009.12.017 20331949

[10] Barbar S Noventa F Rossetto V Ferrari A Brandolin B Perlati M A risk assessment model for the identification of hospitalized medical patients at risk for venous thromboembolism: the Padua Prediction Score. J Thromb Haemost (2010) 8:2450–7. 10.1111/j.1538-7836.2010.04044.x 20738765

[11] Kahn SR Lim W Dunn AS Cushman M Dentali F Akl EA Prevention of VTE in nonsurgical patients: antithrombotic therapy and prevention of thrombosis, 9th ed: American college of chest physicians evidence-based clinical practice guidelines. Chest (2012) 141:195S–226S. 10.1378/chest.11-2296 PMC3278052 22315261

[12] Goudie A Dynan L Brady PW Fieldston E Brilli RJ Walsh KE. Costs of venous thromboembolism, catheter-associated urinary tract infection, and pressure ulcer. Pediatrics (2015) 136:432–9. 10.1542/peds.2015-1386 26260712

[13] Mahajerin A Branchford BR Amankwah EK Raffini L Chalmers E van Ommen CH Hospital-associated venous thromboembolism in pediatrics: a systematic review and meta-analysis of risk factors and risk-assessment models. Haematologica (2015) 100:1045–50. 10.3324/haematol.2015.123455 26001789 PMC5004420

[14] Setty BA O’Brien SH Kerlin BA. Pediatric venous thromboembolism in the United States: a tertiary care complication of chronic diseases. Pediatr Blood Cancer (2012) 59:258–64. 10.1002/pbc.23388 22038730 PMC3270120

[15] Ye F Stalvey C Khuddus MA Winchester DE Toklu HZ Mazza JJ A systematic review of mobility/immobility in thromboembolism risk assessment models for hospitalized patients. J Thromb Thrombolysis (2017) 44:94–103. 10.1007/s11239-017-1501-5 28484939

[16] Nendaz M Spirk D Kucher N Aujesky D Hayoz D Beer JH Multicentre validation of the Geneva Risk Score for hospitalised medical patients at risk of venous thromboembolism. Explicit ASsessment of Thromboembolic RIsk and Prophylaxis for Medical PATients in SwitzErland (ESTIMATE). Thromb Haemost (2014) 111:531–8. 10.1160/TH13-05-0427 24226257

[17] Gould MK Garcia DA Wren SM Karanicolas PJ Arcelus JI Heit JA Prevention of VTE in nonorthopedic surgical patients: antithrombotic therapy and prevention of thrombosis, 9th ed: American college of chest physicians evidence-based clinical practice guidelines. Chest (2012) 141:e227S 10.1378/chest.11-2297 22315263 PMC3278061

[18] Branchford BR Mourani P Bajaj L Manco-Johnson M Wang M Goldenberg NA. Risk factors for in-hospital venous thromboembolism in children: a case-control study employing diagnostic validation. Haematologica (2012) 97:509–15. 10.3324/haematol.2011.054775 22133768 PMC3347665

[19] Arlikar SJ Atchison CM Amankwah EK Ayala IA Barrett LA Branchford BR Development of a new risk score for hospital-associated venous thromboembolism in critically-ill children not undergoing cardiothoracic surgery. Thromb Res (2015) 136:717–22. 10.1016/j.thromres.2015.04.036 25979250

[20] Atchison CM Arlikar S Amankwah E Ayala I Barrett L Branchford BR Development of a new risk score for hospital-associated venous thromboembolism in noncritically ill children: findings from a large single-institutional case-control study. J Pediatr (2014) 165:793–8. 10.1016/j.jpeds.2014.05.053 25064163 PMC7269107

[21] Martinod K Wagner DD. Thrombosis: tangled up in NETs. Blood (2014) 123:2768–76. 10.1182/blood-2013-10-463646 24366358 PMC4007606

[22] Stokes S Breheny P Radulescu A Radulescu VC. Impact of obesity on the risk of venous thromboembolism in an inpatient pediatric population. Pediatr Hematol Oncol (2014) 31:475–80. 10.3109/08880018.2014.886315 24684263 PMC4410870

[23] Jain A Karas DJ Skolasky RL Sponseller PD. Thromboembolic complications in children after spinal fusion surgery. Spine (Phila Pa 1976) (2014) 39:1325–9. 10.1097/BRS.0000000000000402 25010014

[24] Greenwald LJ Yost MT Sponseller PD Abdullah F Ziegfeld SM Ain MC. The role of clinically significant venous thromboembolism and thromboprophylaxis in pediatric patients with pelvic or femoral fractures. J Pediatr Orthop (2012) 32:357–61. 10.1097/BPO.0b013e31824b2a07 22584835

[25] Manlhiot C Menjak IB Brandao LR Gruenwald CE Schwartz SM Sivarajan VB Risk, clinical features, and outcomes of thrombosis associated with pediatric cardiac surgery. Circulation (2011) 124:1511–9. 10.1161/CIRCULATIONAHA.110.006304 21911785

[26] Schroeder AR Axelrod DM Silverman NH Rubesova E Merkel E Roth SJ A continuous heparin infusion does not prevent catheter-related thrombosis in infants after cardiac surgery. Pediatr Crit Care Med (2010) 11:489–95. 10.1097/PCC.0b013e3181ce6e29 20101197

[27] Van Arendonk KJ Schneider EB Haider AH Colombani PM Stewart FD Haut ER. Venous thromboembolism after trauma: when do children become adults? JAMA Surg (2013) 148:1123–30. 10.1001/jamasurg.2013.3558 24173244

[28] Yen J Van Arendonk KJ Streiff MB McNamara L Stewart FD Conner KG Risk factors for venous thromboembolism in pediatric trauma patients and validation of a novel scoring system: the risk of clots in kids with trauma score. Pediatr Crit Care Med (2016) 17:391–9. 10.1097/PCC.0000000000000699 26963757 PMC5270614

[29] Kanin M Young G. Incidence of thrombosis in children with tunneled central venous access devices versus peripherally inserted central catheters (PICCs). Thromb Res (2013) 132:527–30. 10.1016/j.thromres.2013.08.018 24055175

[30] Smitherman AB Alexander T Connelly M Snavely AC Weston BW Liles EA The incidence of catheter-associated venous thrombosis in noncritically ill children. Hosp Pediatr (2015) 5:59–66. 10.1542/hpeds.2014-0041 25646197

[31] Beck C Dubois J Grignon A Lacroix J David M. Incidence and risk factors of catheter-related deep vein thrombosis in a pediatric intensive care unit: a prospective study. J Pediatr (1998) 133:237–41. 10.1016/S0022-3476(98)70226-4 9709712

[32] Lamperti M Bodenham AR Pittiruti M Blaivas M Augoustides JG Elbarbary M International evidence-based recommendations on ultrasound-guided vascular access. Intensive Care Med (2012) 38:1105–17. 10.1007/s00134-012-2597-x 22614241

[33] Kenet G Lutkhoff LK Albisetti M Bernard T Bonduel M Brandao L Impact of thrombophilia on risk of arterial ischemic stroke or cerebral sinovenous thrombosis in neonates and children: a systematic review and meta-analysis of observational studies. Circulation (2010) 121:1838–47. 10.1161/CIRCULATIONAHA.109.913673 20385928

[34] Mitchell L Lambers M Flege S Kenet G Li-Thiao-Te V Holzhauer S Validation of a predictive model for identifying an increased risk for thromboembolism in children with acute lymphoblastic leukemia: results of a multicenter cohort study. Blood (2010) 115:4999–5004. 10.1182/blood-2010-01-263012 20339086 PMC2890143

[35] Sharathkumar AA Mahajerin A Heidt L Doerfer K Heiny M Vik T Risk-prediction tool for identifying hospitalized children with a predisposition for development of venous thromboembolism: Peds-Clot clinical Decision Rule. J Thromb Haemost (2012) 10:1326–34. 10.1111/j.1538-7836.2012.04779.x 22583578

[36] Chopard P Spirk D Bounameaux H Identifying acutely ill medical patients requiring thromboprophylaxis. J Thromb Haemost (2006) 4:915–6. 10.1111/j.1538-7836.2006.01818.x 16634771

[37] Spyropoulos AC Anderson FA Jr Fitzgerald G Decousus H Pini M Chong BH Predictive and associative models to identify hospitalized medical patients at risk for VTE. Chest (2011) 140:706–14. 10.1378/chest.10-1944 21436241

[38] Connelly CR Laird A Barton JS Fischer PE Krishnaswami S Schreiber MA A clinical tool for the prediction of venous thromboembolism in pediatric trauma patients. JAMA Surg (2016) 151:50–7. 10.1001/jamasurg.2015.2670 26422678

[39] Nowak-Gottl U Bidlingmaier C Krumpel A Gottl L Kenet G. Pharmacokinetics, efficacy, and safety of LMWHs in venous thrombosis and stroke in neonates, infants and children. Br J Pharmacol (2008) 153:1120–7. 10.1038/sj.bjp.0707447 17906688 PMC2275453

[40] Raffini L Trimarchi T Beliveau J Davis D. Thromboprophylaxis in a pediatric hospital: a patient-safety and quality-improvement initiative. Pediatrics (2011) 127:e1326–32. 10.1542/peds.2010-3282 21464186

[41] Stem J Christensen A Davis D Raffini L. Safety of prophylactic anticoagulation at a pediatric hospital. J Pediatr Hematol Oncol (2013) 35:e287–91. 10.1097/MPH.0b013e31829b7f92 23774158

[42] Martinez-Velilla N Cadore L Casas-Herrero A Idoate-Saralegui F Izquierdo M Physical activity and early rehabilitation in hospitalized elderly medical patients: systematic review of randomized clinical trials. J Nutr Health Aging (2016) 20:738–51. 10.1007/s12603-016-0683-4 27499308 PMC12879149

[43] Pashikanti L Von Ah D. Impact of early mobilization protocol on the medical-surgical inpatient population: an integrated review of literature. Clin Nurse Spec (2012) 26:87–94. 10.1097/NUR.0b013e31824590e6 22336934

[44] Allenby F Boardman L Pflug JJ Calnan JS Effects of external pneumatic intermittent compression on fibrinolysis in man. Lancet (1973) 2:1412–4. 10.1016/S0140-6736(73)92802-X 4128724

[45] Cahan MA Hanna DJ Wiley LA Cox DK Killewich LA. External pneumatic compression and fibrinolysis in abdominal surgery. J Vasc Surg (2000) 32:537–43. 10.1067/mva.2000.107572 10957661

[46] Comerota AJ Chouhan V Harada RN Sun L Hosking J Veermansunemi R The fibrinolytic effects of intermittent pneumatic compression: mechanism of enhanced fibrinolysis. Ann Surg (1997) 226:306–313; discussion 313–304. 10.1097/00000658-199709000-00010 9339937 PMC1191029

[47] Kosir MA Schmittinger L Barno-Winarski L Duddella P Pone M Perales A Prospective double-arm study of fibrinolysis in surgical patients. J Surg Res (1998) 74:96–101. 10.1006/jsre.1997.5233 9536981

[48] Macaulay W Westrich G Sharrock N Sculco TP Jhon PH Peterson MG Effect of pneumatic compression on fibrinolysis after total hip arthroplasty. Clin Orthop Relat Res (2002) 399:168–76. 10.1097/00003086-200206000-00020 12011706

[49] O’Brien TE Woodford M Irving MH. The effect of intermittent compression of the calf on the fibrinolytic responses in the blood during a surgical operation. Surg Gynecol Obstet (1979) 149:380–4. 473000

[50] Tarnay TJ Rohr PR Davidson AG Stevenson MM Byars EF Hopkins GR. Pneumatic calf compression, fibrinolysis, and the prevention of deep venous thrombosis. Surgery (1980) 88:489–96. 7423372

[51] Arabi YM Khedr M Dara SI Dhar GS Bhat SA Tamim HM Use of intermittent pneumatic compression and not graduated compression stockings is associated with lower incident VTE in critically ill patients: a multiple propensity scores adjusted analysis. Chest (2013) 144:152–9. 10.1378/chest.12-2028 23412593

[52] Barrera LM Perel P Ker K Cirocchi R Farinella E Morales Uribe CH. Thromboprophylaxis for trauma patients. Cochrane Database Syst Rev (2013) 3:CD008303. 10.1002/14651858.CD008303.pub2 23543562 PMC12107512

[53] Handoll HH Farrar MJ McBirnie J Tytherleigh-Strong G Milne AA Gillespie WJ Heparin, low molecular weight heparin and physical methods for preventing deep vein thrombosis and pulmonary embolism following surgery for hip fractures. Cochrane Database Syst Rev (2002) 4:CD000305 10.1002/14651858.CD000305 12519540

[54] Ho KM Tan JA. Stratified meta-analysis of intermittent pneumatic compression of the lower limbs to prevent venous thromboembolism in hospitalized patients. Circulation (2013) 128:1003–20. 10.1161/CIRCULATIONAHA.113.002690 23852609

[55] Kakkos SK Caprini JA Geroulakos G Nicolaides AN Stansby GP Tsolakis IA Can combined (mechanical and pharmacological) modalities prevent fatal VTE? Int Angiol (2011) 30:115–22. 21427647

[56] Monagle P Chan AK Goldenberg NA Ichord RN Journeycake JM Nowak-Gottl U Antithrombotic therapy in neonates and children: antithrombotic therapy and prevention of thrombosis, 9th ed: American college of chest physicians evidence-based clinical practice guidelines. Chest (2012) 141:737S–801S. 10.1378/chest.11-2308 22315277 PMC3278066

[57] Massicotte P Julian JA Gent M Shields K Marzinotto V Szechtman B An open-label randomized controlled trial of low molecular weight heparin for the prevention of central venous line-related thrombotic complications in children: the PROTEKT trial. Thromb Res (2003) 109:101–8. 10.1016/S0049-3848(03)00059-8 12706638

[58] Ruud E Holmstrom H De Lange C Hogstad EM Wesenberg F Low-dose warfarin for the prevention of central line-associated thromboses in children with malignancies – a randomized, controlled study. Acta Paediatr (2006) 95:1053–9. 10.1080/08035250600729092 16938749

[59] Vidal E Sharathkumar A Glover J Faustino EV. Central venous catheter-related thrombosis and thromboprophylaxis in children: a systematic review and meta-analysis. J Thromb Haemost (2014) 12:1096–109. 10.1111/jth.12598 24801495 PMC4107177

[60] Jaffray J Witmer C Pediatric venous thromboembolism. 1st ed In: Dandoy CE Hilden JM Billett AL Mueller BU, editors. Patient Safety and Quality in Pediatric Hematology/Oncology and Stem Cell Transplantation. Cham: Springer International Publishing (2017). p. 205–25.

[61] Kearon C. Diagnosis of suspected venous thromboembolism. Hematology Am Soc Hematol Educ Program (2016) 2016:397–403. 10.1182/asheducation-2016.1.397 27913507 PMC6142443

[62] Goldenberg NA Knapp-Clevenger R Manco-Johnson MJ Mountain States Regional Thrombophilia Group Elevated plasma factor VIII and d-dimer levels as predictors of poor outcomes of thrombosis in children. N Engl J Med (2004) 351:1081–8. 10.1056/NEJMoa040161 15356305

